# BSG Isoform 2 (ENST00000353555) Is a Better Component Than Total BSG Expression in Generating Prognostic Signature for Overall Survival of Liver Cancer

**DOI:** 10.7759/cureus.62287

**Published:** 2024-06-13

**Authors:** Wei Xiong, Ying Deng

**Affiliations:** 1 Department of Hepatobiliary Surgery, Sichuan Provincial People's Hospital, Chengdu, CHN; 2 Cancer Center, Sichuan Provincial People's Hospital, Chengdu, CHN

**Keywords:** vegfa, overall survival, liver cancer, enst00000353555, bsg

## Abstract

Background: The basigin (BSG) gene, also known as CD147, has been implicated in the progression and prognosis of various cancers, including liver cancer. This study aimed to comprehensively evaluate the prognostic value of total BSG expression and its specific transcript variants, ENST00000353555 and ENST00000545507, in a large cohort of patients with primary liver cancer.

Materials and methods: The prognostic values of total BSG, ENST00000353555, and ENST00000545507 expression in overall survival (OS) and progression-free interval (PFI) of patients with primary liver cancer were assessed using The Cancer Genome Atlas Liver Hepatocellular Carcinoma (TCGA-LIHC) dataset. Survival analysis, receiver operating characteristic (ROC) analysis, and validation of an extracellular matrix (ECM)-related prognostic signature were performed.

Results: In univariate and multivariate analyses, total BSG, ENST00000353555, and ENST00000545507 expression were associated with poor OS in liver cancer patients. ENST00000353555 showed the highest hazard ratio among the three prognostic indicators. ROC analysis revealed that ENST00000353555 had better prognostic performance than total BSG expression. Replacing total BSG with ENST00000353555 in an existing ECM-related prognostic signature marginally increased the area under the curve values for one year from 0.79 to 0.80, and five-year OS from 0.72 to 0.73. ENST00000353555 showed isoform-specific positive correlations with EDNRB, IL10, C10orf54, and VEGFA.

Conclusions: ENST00000353555 serves as a better prognostic biomarker than total BSG expression in liver cancer, either as an individual marker or as a component of an ECM-related gene signature. Additionally, ENST00000353555 exhibited isoform-specific positive correlations with several immunosuppressive genes, suggesting a potential role in regulating the tumor microenvironment.

## Introduction

Liver cancer is a leading cause of cancer-related deaths worldwide, with a five-year survival rate below 20% [1]. Identifying reliable prognostic biomarkers is crucial for improving risk stratification and treatment management for patients with liver cancer. The basigin gene (BSG), also known as CD147, has been implicated in the progression and prognosis of various cancers, including liver cancer [2,3]. Recent studies have highlighted the role of BSG isoforms in cancer biology, suggesting that individual transcript variants may have distinct functional characteristics and prognostic significance. Mechanistically, BSG overexpression can induce angiogenesis, activate matrix metalloproteinases to degrade extracellular matrix, promote cancer cell invasion, and inhibit cancer cell anoikis (cell death after detachment) [4,5].

High BSG expression is also associated with poor prognosis in hepatocellular carcinoma patients [2,6]. However, the discriminative ability of the prediction model based on a single gene is usually not satisfactory [7]. To improve the prognostic sensitivity and specificity, BSG expression can be utilized with other genes to form a gene expression signature for prognosis estimation [8,9]. Alternative splicing and different transcriptional start sites are key post-transcriptional regulatory processes that are crucial in generating transcriptomic and proteomic diversity in human cells. More than 95% of human genes are estimated to undergo alternative splicing, resulting in structural variation of transcripts and an expansion of the proteome [10,11]. Current knowledge indicates that BSG can give rise to three distinct protein isoforms encoded by four mRNA transcript variants. These include NM_001728.4/ENST00000333511.9 encoding basigin-1, NM_198589.3/ENST00000353555.9 encoding basigin-2, and NM_198590.3/ENST00000545507.6 and NM_198591.4/ENST00000346916.9 encoding basigin-3. These individual BSG isoforms have been found to exert distinct regulatory effects in the context of cancer [12-14].

In this study, we aimed to comprehensively evaluate the prognostic value of total BSG expression and its specific transcript variants, ENST00000353555 and ENST00000545507, in a large cohort of patients with primary liver cancer from The Cancer Genome Atlas (TCGA) database. We further investigated the potential of using ENST00000353555 as an improved prognostic biomarker, either as an individual marker or as a component of an extracellular matrix (ECM)-related gene signature. Additionally, we explored the isoform-specific associations between BSG transcripts and key immunosuppressive genes in the liver tumor microenvironment.

## Materials and methods

Data extraction and survival analysis

The prognostic values of total BSG, ENST00000353555, and ENST00000545507 expression in overall survival (OS) and progression-free interval (PFI) of patients with primary liver cancer were assessed using the TCGA-LIHC dataset (which are publicly accessible and de-identified), based on data obtained from the University of California, Santa Cruz (UCSC) database (https://xenabrowser.net/) [15]. The gene expression and transcript-specific expression data were log2-transformed (TPM+0.001) to prepare the input for further analyses. The clinical outcome data, including overall survival (OS) and progression-free interval (PFI), were extracted for the survival analysis. The final dataset included 363 primary liver cancer cases with OS information and 364 cases with PFI data.

Survival analysis

The association between gene or transcript expression (as a continuous variable) and patient prognosis (OS and PFI) was assessed using the Cox proportional hazards regression model [16]. The analysis was performed using the survival package (version 3.5-7) in the R software (R Foundation, Vienna, Austria) environment.

Receiver operating characteristic (ROC) analysis

ROC analysis was performed using the R software package "pROC" (version 1.17.0.1) (R Foundation, Vienna, Austria) to obtain the area under the ROC Curve (AUC). Specifically, the "roc" function of pROC to conduct ROC analysis of total BSG or ENST00000353555 for OS detection at time points of 365, 1095, and 1825 days. The AUC and confidence intervals were evaluated using the "ci" function of pROC to obtain the final AUC results.

Validation and improvement of the ECM-related prognostic signature

The extracellular matrix (ECM)-related six-gene prognostic signature containing BSG [8] was improved by replacing total BSG with ENST00000353555 expression. The R software package Glmnet (R Foundation, Vienna, Austria) was utilized to integrate overall survival time, overall survival status, and gene or transcript expression data for regression analysis, using the least absolute shrinkage and selection operator (LASSO) regression method. A 10-fold cross-validation was further conducted to obtain the optimal model (the optimal lambda value).

Statistical analysis

Pearson’s r values were calculated for correlations. Univariate and multivariate Cox regression analysis was conducted to assess the independent prognostic value of ENST00000353555; p<0.05 was considered statistically significant.

## Results

Comparison of the prognostic values of total BSG, ENST00000353555, and ENST00000545507 expression in liver cancer

**Figure 1:**
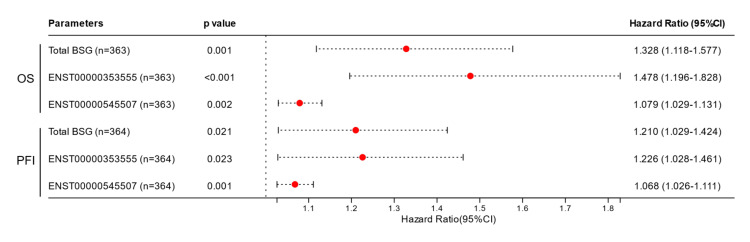
Comparison of the prognostic values of total BSG, ENST00000353555, and ENST00000545507 expression in liver cancer A forest plot chart was generated to show the univariate Cox regression analysis of total BSG, ENST00000353555 and ENST00000545507 expression in OS or PFI prediction in patients with primary liver cancer in TCGA.

Based on gene expression and survival data in TCGA-LIHC, we assessed the prognostic values of total BSG, ENST00000353555, and ENST00000545507 expression in OS and PFI in patients with primary liver cancer. In univariate analysis, total BSG, ENST00000353555, and ENST00000545507 expression were associated with poor OS and PFI in patients with liver cancer (Figure 1, Table 1).

**Table 1:** Univariate analysis of OS in TCGA-LIHC. OS: Overall survival; HR: Hazard ratio, AJCC: American Joint Committee on Cancer; TCGA-LIHC: The Cancer Genome Atlas Liver Hepatocellular Carcinoma.

Univariate analysis for OS				
Parameters	p value	HR	95%CI	
Age (n=363)	0.094	1.012	0.998	1.026
Gender	-	-	-	-
Male (n=245)	-	1.0000	-	-
Female (n=118)	0.289	1.213	0.849	1.734
AJCC stages	-	-	-	-
III/IV (n=87)	-	1.0000	-	-
I/II (n=252)	<0.001	0.391	0.269	0.568
Histological grade	-	-	-	-
G4 (n=11)	-	1.0000	-	-
G1 (n=55)	0.500	0.688	0.232	2.042
G2 (n=175)	0.685	0.810	0.293	2.238
G3 (n=117)	0.723	0.830	0.297	2.322
Gene or transcript expression	-	-	-	-
Total BSG (n=363)	0.001	1.328	1.118	1.577
ENST00000353555 (n=363)	<0.001	1.478	1.196	1.828
ENST00000545507 (n=363)	0.002	1.079	1.029	1.131

Multivariate Cox regression analysis confirmed the independent prognostic significance of total BSG, ENST00000353555, and ENST00000545507 expression, after adjustment of American Joint Committee on Cancer (AJCC) stages (Table 2). However, ENST00000353555 has the highest hazard ratio (HR) values (Table 2).

**Table 2:** Multivariate analysis of OS in TCGA-LIHC OS: Overall survival; AJCC: American Joint Committee on Cancer; TCGA-LIHC: The Cancer Genome Atlas Liver Hepatocellular Carcinoma.

Gene or transcript expression	Multivariate analysis for OS (after adjustment for AJCC stages)					
Total BSG (n=363)		0.002	1.337	1.117	1.599	
ENST00000353555 (n=363)		0.001	1.424	1.151	1.762	
ENST00000545507 (n=363)		0.006	1.068	1.019	1.119	

ENST00000353555 serves as a better prognostic biomarker than total BSG expression, either as an individual biomarker or a component of a prognostic signature

**Figure 2:**
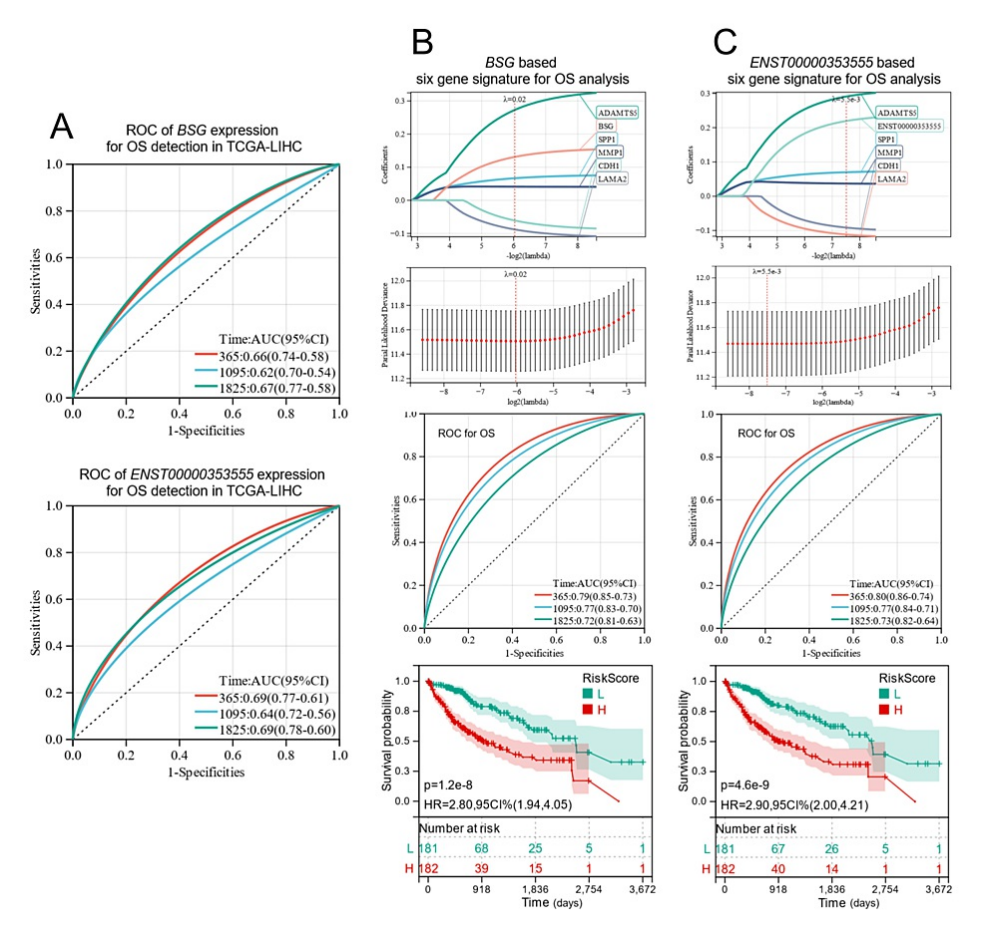
ENST00000353555 serves as a better prognostic biomarker than total BSG expression either as an individual biomarker or a component of a prognostic signature. A-B. The ROC curves and AUC values of total BSG (A) and ENST00000353555 (B) for OS detection at time points of 365, 1095, and 1825 days among patients with primary liver cancer in TCGA-LIHC. B-C. Validation of the BSG-based extracellular matrix-related prognostic signature (B) and the improved signature with ENST00000353555 (C). Panels from the top to the bottom: Least absolute shrinkage and selection operator (LASSO) coefficient profiles of the prognostic genes (top). Parameter selection in the LASSO model (second from the top). The ROC curves and AUC values of the signature for OS detection at time points of 365, 1095, and 1825 days among patients with primary liver cancer in TCGA-LIHC (third from the top). Kaplan-Meier survival curves for OS comparisons were generated by dividing patients with primary liver cancer in TCGA-LIHC into two groups based on the median signature scores (50%) (bottom). A log-rank test was performed to analyze the statistical significance between the higher and lower expression groups. OS: Overall survival; ROC: Receiver operating characteristic; AUC: Area under the curve; TCGA-LIHC: The Cancer Genome Atlas Liver Hepatocellular Carcinoma; HR: Hazard ratio; CI: Confidence interval.

Since ENST00000353555 has higher HR values than total BSG in OS prediction, we hypothesized that ENST00000353555 might be a better prognostic biomarker than total BSG expression. To validate this hypothesis, we conducted ROC analysis using total BSG or ENST00000353555 expression for OS detection at 365, 1095, and 1825 days. For total BSG, the AUC values at each time point were 0.66, 0.62, and 0.67 (Figure 2A). However, the AUC values increased to 0.69, 0.64, and 0.69 when we replaced the total BSG with ENST00000353555 expression (Figure 2B). Although the AUC value nearly reached 0.7, which means moderate accuracy in outcomes, this level of AUC is not good enough for a prognostic biomarker [17,18]. One recent study found that BSG, together with five other extracellular matrix-related genes (SPP1, ADAMTS5, MMP1, LAMA2, and CDH1) can form a signature with much improved AUC values [8]. The AUC values of this signature at 365, 1095, and 1825 days were 0.79, 0.77, and 0.72, respectively [8]. We validated these findings and confirmed a substantial OS difference (HR: 2.80, 95%CI: 1.94-4.05, p<0.001) between the groups with higher signature scores and lower signature scores (Figure 2B, bottom panel). If we replace BSG with ENST00000353555, the following new signature risk scores could be generated: RiskScore=0.0700137019893281*SPP1+0.290867111936407*ADAMTS5+0.0369815297118727*MMP1-0.111714850094493*LAMA2-0.0915232748912096*CDH1+0.219431477220605*ENST00000353555. The AUC value of this model further increased to 0.80, 0.77 and 0.73 respectively (Figure 2C), with greater OS difference between the groups with higher signature scores and lower signature scores (HR: 2.90, 95%CI: 2.00-4.21, p<0.001) (Figure 2C, bottom panel).

Total BSG and ENST00000353555 have great differences in the expressional correlation with multiple immune-suppressive genes

**Figure 3:**
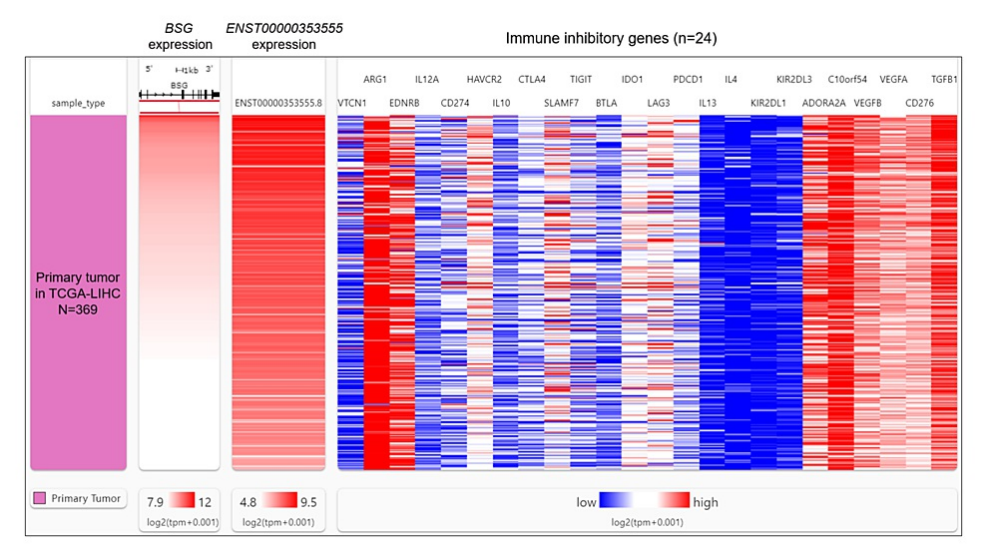
ENST00000353555 showed isoform-specific positive correlations with EDNRB, IL10, C10orf54, and VEGFA. A heatmap showing the correlation of total BSG expression and ENST00000353555 with 24 immunosuppressive genes, including VTCN1, ARG1, EDNRB, IL12A, CD274, HAVCR2, IL10, CTLA4, SLAMF7, TIGIT, BTLA, IDO1, LAG3, PDCD1, IL13, IL4, KIR2DL1, KIR2DL3, ADORA2A, C10orf54, VEGFB, VEGFA, CD276, and TGFB1, in primary liver cancer cases in TCGA-LIHC. TCGA-LIHC: The Cancer Genome Atlas Liver Hepatocellular Carcinoma.

Previous studies reported that BSG could enhance malignant tumor behaviors via immunosuppression in the liver tumor microenvironment [19,20]. To explore whether ENST00000353555 exerts isoform-specific regulation on the immunosuppression, we compared the correlation of total BSG expression and ENST00000353555 with 24 immunosuppressive genes identified in a previous publication [21], including VTCN1, ARG1, EDNRB, IL12A, CD274, HAVCR2, IL10, CTLA4, SLAMF7, TIGIT, BTLA, IDO1, LAG3, PDCD1, IL13, IL4, KIR2DL1, KIR2DL3, ADORA2A, C10orf54, VEGFB, VEGFA, CD276, and TGFB1, in primary liver cancer cases in TCGA-LIHC (Figure 3).

The correlation coefficients were calculated individually. The genes with differences larger than 0.2 in the correlation coefficients were identified. ENST00000353555 showed isoform-specific positive correlations with EDNRB, IL10, C10orf54, and VEGFA (Table 3).

**Table 3:** The correlation coefficients of total BSG expression and ENST00000353555 with the 24 immunosuppressive genes.

Immune inhibitory genes	Correlation with BSG	Correlation with ENST00000353555	Discrepancy
VTCN1	0.24	0.30	0.06
ARG1	-0.34	-0.20	0.13
EDNRB	-0.16	0.21	0.37
IL12A	0.12	0.19	0.07
CD274	0.16	0.33	0.17
HAVCR2	0.20	0.26	0.06
IL10	0.01	0.22	0.21
CTLA4	0.18	0.12	-0.06
SLAMF7	0.08	0.11	0.03
TIGIT	0.10	0.14	0.03
BTLA	-0.01	0.03	0.04
IDO1	0.02	0.18	0.16
LAG3	0.13	-0.02	-0.14
PDCD1	0.23	0.21	-0.02
IL13	0.03	0.12	0.09
IL4	0.07	0.05	-0.02
KIR2DL1	-0.01	0.12	0.13
KIR2DL3	-0.01	0.13	0.14
ADORA2A	0.17	0.32	0.16
C10orf54	0.06	0.30	0.24
VEGFB	0.33	0.19	-0.14
VEGFA	0.18	0.51	0.33
CD276	0.41	0.43	0.02
TGFB1	0.37	0.35	-0.03

## Discussion

Although previous studies have confirmed that BSG expression is an unfavorable prognostic biomarker in liver cancer [3,22,23], transcript-specific prognosis has not yet been explored. In this study, we demonstrated that ENST00000353555 has a better prognostic value than total BSG expression, either as an individual biomarker or a component of a prognostic signature. By replacing total BSG expression with ENST00000353555 in an established ECM-gene-based prognostic signature in liver cancer [8], it marginally increased the AUC values for one year from 0.79 to 0.80, and five-year OS from 0.72 to 0.73. In clinical use, an AUC improvement by 0.01 could slightly enhance risk stratification capacity for patients. Although the improvement might be marginal, it indicates an improvement direction. For genes encoding multiple protein isoforms, transcript-specific prognosis prediction might improve sensitivity and specificity. Moreover, replacing total gene expression with a gene-specific transcript in the signature might have other implications beyond improving the AUC. For example, it could lead to a better understanding of the disease's molecular mechanisms, or it could make the signature easier to use in clinical practice by reducing the number of genes that need to be measured.

In this study, ENST00000353555 showed isoform-specific positive correlations with EDNRB, IL10, C10orf54, and VEGFA. EDNRB (endothelin receptor type B) has been shown to promote immunosuppression in hepatocellular carcinoma. EDNRB activation inhibits natural killer cell function and promotes the accumulation of immunosuppressive cells like regulatory T cells and myeloid-derived suppressor cells in the tumor microenvironment [24]. This allows cancer cells to evade immune detection. Higher EDNRB expression correlates with reduced immune cell infiltration [24] and poorer prognosis in hepatocellular carcinoma patients [25]. IL10 (interleukin 10) is an anti-inflammatory cytokine that exerts immunosuppressive effects in liver cancer. It inhibits the activation and effector functions of T cells, NK cells, dendritic cells, and macrophages. It also promotes the accumulation of immunosuppressive regulatory T cells [26]. These mechanisms help create an immunosuppressive tumor microenvironment that facilitates immune escape. High IL10 expression has been associated with tumor progression and poor survival in hepatocellular carcinoma patients [27]. C10orf54, also known as VISTA (V-domain Ig suppressor of T cell activation), is a novel immune checkpoint molecule [28].

VEGFA (vascular endothelial growth factor A) is a growth factor that plays a crucial role in angiogenesis [29]. It also promotes immunosuppression in hepatocellular carcinoma through multiple mechanisms. It inhibits the maturation and activation of dendritic cells, impairs T cell development, and recruits immunosuppressive regulatory T cells and myeloid cells like tumor-associated macrophages [30]. High VEGFA expression is also associated with decreased tumor-infiltrating lymphocytes and poorer prognosis in hepatocellular carcinoma patients. It’s known that BSG can stimulate the expression of VEGFA via the PI3K-Akt signaling pathway [31-33]. In this study, we observed that Pearson’s r is 0.51 (moderate positive correlation) between ENST00000353555 and VEGFA but is only 0.18 (no correlation) between total BSG and VEGFA. Therefore, it is reasonable to infer that ENST00000353555 has a causative effect on VEGFA overexpression. These mechanisms suggest that ENST00000353555 might exert isoform-specific immunosuppressive effects in the tumor microenvironment of liver cancer. ENST00000353555 specific knockdown might generate better tumor suppressive effects than total BSG depletion in liver cancer. These results underscore the superior prognostic value of ENST00000353555 over total BSG expression.

However, the limitations of this study, including its retrospective nature and reliance on a single dataset, should be acknowledged. Future prospective studies are necessary to validate these findings and explore the mechanistic pathways through which ENST00000353555 influences liver cancer prognosis.

## Conclusions

ENST00000353555 has a better prognostic value than total BSG expression in liver cancer, either as an individual biomarker or a component of a prognostic signature. ENST00000353555 might have a transcript-specific causative effect on the expression of multiple immunosuppressive genes, such as EDNRB, IL10, C10orf54, and VEGFA. However, future molecular studies are required for validation.
